# The Temporal Dynamics of EEG Microstate Reveals the Neuromodulation Effect of Acupuncture With *Deqi*

**DOI:** 10.3389/fnins.2021.715512

**Published:** 2021-10-07

**Authors:** Xiaopeng Si, Shunli Han, Kuo Zhang, Ludan Zhang, Yulin Sun, Jiayue Yu, Dong Ming

**Affiliations:** ^1^Academy of Medical Engineering and Translational Medicine, Tianjin University, Tianjin, China; ^2^Tianjin Key Laboratory of Brain Science and Neural Engineering, Tianjin University, Tianjin, China; ^3^Tianjin International Engineering Institute, Tianjin University, Tianjin, China; ^4^Institute of Applied Psychology, Tianjin University, Tianjin, China

**Keywords:** acupuncture, *deqi*, temporal dynamics, microstate parameter, translation probability, microstate C, microstate D

## Abstract

The electroencephalography (EEG) microstate has recently emerged as a new whole-brain mapping tool for studying the temporal dynamics of the human brain. Meanwhile, the neuromodulation effect of external stimulation on the human brain is of increasing interest to neuroscientists. Acupuncture, which originated in ancient China, is recognized as an external neuromodulation method with therapeutic effects. Effective acupuncture could elicit the *deqi* effect, which is a combination of multiple sensations. However, whether the EEG microstate could be used to reveal the neuromodulation effect of acupuncture with *deqi* remains largely unclear. In this study, multichannel EEG data were recorded from 16 healthy subjects during acupuncture manipulation, as well as during pre- and post-manipulation tactile controls and pre- and post-acupuncture rest controls. As the basic acupuncture unit for regulating the central nervous system, the Hegu acupoint was used in this study, and each subject’s acupuncture *deqi* behavior scores were collected. To reveal the neuroimaging evidence of acupuncture with *deqi*, EEG microstate analysis was conducted to obtain the microstate maps and microstate parameters for different conditions. Furthermore, Pearson’s correlation was analyzed to investigate the correlation relationship between microstate parameters and *deqi* behavioral scores. Results showed that: (1) compared with tactile controls, acupuncture manipulation caused significantly increased *deqi* behavioral scores. (2) Acupuncture manipulation significantly increased the duration, occurrence, and contribution parameters of microstate C, whereas it decreased those parameters of microstate D. (3) Microstate C’s duration parameter showed a significantly positive correlation with acupuncture *deqi* behavior scores. (4) Acupuncture manipulation significantly increased the transition probabilities with microstate C as node, whereas it reduced the transition probabilities with microstate D as node. (5) Microstate B→C’s transition probability also showed a significantly positive correlation with acupuncture *deqi* behavior scores. Taken together, the temporal dynamic feature of EEG microstate could be used as objective neuroimaging evidence to reveal the neuromodulation effect of acupuncture with *deqi*.

## Introduction

The dynamics and functions of the nervous system could be efficiently modulated by external stimuli, which has received increasing attention from neuroscientists (Marder, 2012). Acupuncture, as an external stimulation to the human nerve system, has been applied to manage various clinical diseases, such as neuropathic pains (Miranda et al., 2015), epilepsy (Nicolaou and Georgiou, 2012), Alzheimer’s disease (Liang et al., 2014), and Parkinson’s disease (Aroxa et al., 2017), which has received increasing attention by the public. Moreover, previous studies showed that acupuncture could modulate the human brain by balancing the parasympathetic and sympathetic systems (Wu et al., 1999; Ernst, 2006; Takahashi, 2011). Although the World Health Organization announced that more than 43 diseases could be treated with acupuncture (Hsu et al., 2011), there is a lack of objective neuroimaging evidence for revealing the neuromodulation effect of acupuncture.

According to traditional Chinese medicine (TCM), a fine needle is inserted into the subcutaneous muscle at specific acupoints during acupuncture. Acupuncture could stimulate the afferent nerve and regulate the central nervous system, in which subjects experience the sensation of acupuncture with *deqi* (Takahashi, 2011). Moreover, acupuncture to achieve the *deqi* effect is called effective acupuncture, in which the *deqi* effect is created by a combination of different unique senses (Hui et al., 2007). And, the *deqi* effect is positively correlated with the therapeutic effect (Takahashi, 2011). Studies have shown that the *deqi* effect is an essential factor in acupuncture’s regulation and treatment of various diseases (Fang et al., 2009; Asghar et al., 2010). However, the current method for evaluating the acupuncture effect mainly relies on the subjective *deqi* behavioral questionnaire (Hui et al., 2007; Yu et al., 2012). Therefore, there lacks a reliable objective neuroimaging evidence to reveal the neuromodulation effects of acupuncture with *deqi*.

To reveal the neural mechanism of acupuncture, functional magnetic resonance imaging (fMRI) and electroencephalograph (EEG) were employed in previous studies (Paraskeva et al., 2004; Onton et al., 2005; Tyvaert et al., 2009). The high-spatial-resolution fMRI studies helped reveal the specific brain areas involved in acupuncture. It was found that acupuncture could activate the insula (Wu et al., 1999; Fang et al., 2009; Chae et al., 2013) and anterior cingulate cortex (Wu et al., 1999; Fang et al., 2009; Chae et al., 2013), whereas it could deactivate the medial prefrontal cortex, caudate, amygdala, and posterior cingulate cortex (Wu et al., 1999; Fang et al., 2009; Chae et al., 2013). These brain response patterns indicated that acupuncture could modulate cognitive functions such as pain perception, memory, and emotional dimensions (Fang et al., 2009; Chae et al., 2013). However, due to fMRI’s poor temporal resolution, fMRI studies failed to reveal the temporal dynamic changes during acupuncture. In addition, studies have found that acupuncture can strengthen brain connectives (Yu et al., 2017, 2018). However, these studies only answer the connectivity between static network nodes, and the study of dynamic characteristics is still unclear. Previous EEG studies mainly focused on the specific frequency band’s neural oscillation changes, in which EEG studies found increased power changes of delta and alpha frequency bands during acupuncture by using frequency analysis methods (Chen et al., 2006; Bian et al., 2011; Yu et al., 2018). Although the high temporal resolution EEG could capture the brain dynamics, previous EEG studies neglected the temporal characteristics during acupuncture. In summary, previous fMRI and EEG neuroimaging studies showed that acupuncture could modulate the neural activities of specific brain areas and regulate the EEG frequency bands. However, previous studies have not revealed the temporal dynamics of the human brain during acupuncture. Therefore, how the human brain is dynamically modulated by acupuncture manipulation remains largely unknown.

The human brain is essentially a dynamic network system (Bassett and Sporns, 2017). The communication within and between large-scale brain networks will fluctuate spontaneously at all times in a well-organized way (Bassett and Sporns, 2017; Kucyi et al., 2017; Hsu et al., 2018). Acupuncture is a way to regulate the neural activity of the brain. Currently, more studies focus on the response of different network nodes and brain regions using fMRI (Wu et al., 1999; Fang et al., 2009; Chae et al., 2013). However, the temporal dynamics of the brain during acupuncture with *deqi* remain unclear. Brain dynamics can be explored by using different neuroimaging methods with different timescales. While the high-spatial-resolution fMRI captures the slow neural dynamics on a second timescale, the faster millisecond timescale neural dynamic changes could be explored by using the EEG microstate analysis (Michel and Koenig, 2018; Schumacher et al., 2019). Previous studies showed that the dynamic patterns of EEG activities are empirically divided into a series of alternating types of quasi-stable EEG topographic maps, which are termed “EEG microstates” (Lehmann et al., 1987; Khanna et al., 2014; Michel and Koenig, 2018). It was proved that there existed four discrete EEG microstates, i.e., microstates A, B, C, and D, which were found to be reproducible across participants (Koenig et al., 2002). The four microstates (A, B, C, and D) showed maximum activity from right frontal to left posterior, left frontal to right posterior, midline frontal occipital, and central frontal (Lehmann et al., 2009). And they are related to four brain networks [auditory network, visual network, salience network, and dorsal attention network (DAN)] (Britz et al., 2010). Each EEG microstate remains stable for a short period of 80–120 ms before rapidly transitioning into another microstate (Lehmann et al., 1987; Khanna et al., 2014; Michel and Koenig, 2018), which could detect the discontinuous and nonlinear dynamic changes of brain activities (Lehmann et al., 1987). Particularly, the EEG microstate has been considered as the basic units of human cognition and was suggested as the “atom of thought,” which gained increasing popularity in recent years (Koenig et al., 1999, 2002; Michel and Koenig, 2018).

Importantly, the parameters of the alternating EEG microstate time series, such as duration, occurrence, and transition probability, were proved to be effective markers both for distinguishing healthy subjects’ different cognition states (Milz et al., 2016) and for detecting the specific brain changes associated with neuropsychiatric diseases (Koenig et al., 1999; Lehmann et al., 2005; Nishida et al., 2013). For example, different mental states, such as different meditation states (Faber et al., 2017) or sleep conditions (Brodbeck et al., 2012; Xu et al., 2020), were found to be correlated with different EEG microstate parameters for healthy subjects. Furthermore, several neuropsychiatric diseases, such as Alzheimer’s disease (Nishida et al., 2013), Parkinson’s disease (Chu et al., 2020), dementia (Schumacher et al., 2019), and schizophrenia (Tomescu et al., 2014; Murphy et al., 2020), could also be characterized by using EEG microstate parameters. Microstates as a way to describe brain dynamics may be affected by acupuncture, thus providing an objective measure of its effect on brain dynamics (Hsu et al., 2018; Abreu et al., 2021). However, to our knowledge, there is no previous research employing the EEG microstate theory to investigate the brain dynamics during acupuncture with *deqi*. Therefore, whether the EEG microstate parameters could be used as markers to reveal the dynamic neuromodulation effects of acupuncture with *deqi* remains largely unclear.

To reveal the temporal dynamics of brain activity during acupuncture with *deqi*, multichannel EEG data were recorded from 16 healthy subjects during acupuncture manipulation, as well as during pre- and post-manipulation tactile controls and pre- and post-acupuncture rest controls. In addition, the Hegu point is one of the most commonly used acupuncture points, located at the midpoint on the radial side of the second metacarpal, which is also known as large intestine 4 (LI4) (Kong et al., 2015). Considering that the Hegu acupoint is the basic neural acupuncture unit that modulates the central nervous system (Zhang et al., 2012), the Hegu point was explored in this study. The subject’s acupuncture *deqi* behavior scores were collected. EEG microstate analysis was conducted to obtain the microstate maps and microstate parameters for different conditions. Furthermore, Pearson’s correlation was analyzed to investigate the correlation relationship between microstate parameters and *deqi* behavior scores. We speculate that the EEG microstate theory could provide a new perspective to help understand the temporal dynamics of the human brain during acupuncture with *deqi*.

## Materials and Methods

### Subjects

Sixteen healthy right-handed participants (24.0 ± 1.9 years old [mean ± SD], age range from 22 to 28 years, 10 males and 6 females) were recruited for this study. All the participants had normal intelligence, and none of the following conditions existed: (a) history of mental illness, (b) brain damage caused by long-term use of drugs, and (c) experience of acupuncture treatment. All participants signed the informed consent approved by the Institutional Review Board and Ethics Committee of Tianjin University. All participants agreed to provide data anonymously. Supplementary Table S1 shows the details of the subjects.

### Acupuncture Experiment Paradigm

Each subject was required to sit in a comfortable chair during the entire process of the acupuncture experiment in a bright and quiet room. Throughout the acupuncture experiment, subjects were asked to keep awake and relaxed during the preparation stage. All acupunctures were performed by an experienced acupuncturist. Once the subject is found to be uncomfortable or not awake, the subject will be timely reminded and asked about the situation.

During the preparation stage, each subject was asked to be familiar with the visual analogue scale (VAS) behavior questionnaire for evaluating the acupuncture *deqi* sensations, which includes the following six sensations: soreness, numbness, distention, heaviness, spread, and dull pain according to the TCM (Hui et al., 2007; Yu et al., 2012). The intensity of each sensation is rated by a point, ranging from 0 to 10 (0–3 mild, 4–6 moderate, 7–8 strong, 9 severe, and 10 intolerable), on a continuous horizontal line on the VAS questionnaire (Lukacz et al., 2004; Hui et al., 2007). Considering that the unique feeling of *deqi* appears through the combination of different detailed sensations (soreness, numbness, distention, heaviness, spread, and dull pain), the comprehensive index could comprehensively characterize the *deqi* effect (Hui et al., 2007).

**FIGURE 1:**
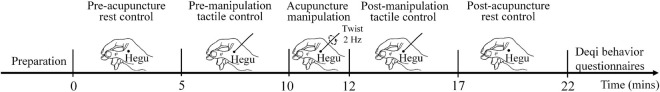
Acupuncture experiment paradigm. During the preparation stage, each subject was asked to be familiar with the behavior questionnaire. There were five conditions in the experiment: 5-min pre-acupuncture rest control, 5-min pre-manipulation tactile control, 2-min acupuncture manipulation, 5-min post-manipulation tactile control, and 5-min post-acupuncture rest control. After the experiment, subjects were asked to evaluate the needling sensations for the pre-manipulation tactile control, acupuncture manipulation, and post-manipulation tactile control. The twisting frequency in the acupuncture manipulation was 2 Hz.

Considering that the quasi-experimental design could compare the condition difference before and after time (Baernighausen et al., 2017), the quasi-experimental design was used in this experiment (Figure 1), which was in line with previous acupuncture researches (Yu et al., 2017, 2018). The whole acupuncture experiment included the following five stages: (I) pre-acupuncture rest control: each subject was asked to maintain awake and rest for 5 min without movement before the needle was inserted by the acupuncturist. (II) Pre-manipulation tactile control: a single-use sterile acupuncture needle was inserted by the acupuncturist at the Hegu acupoint to a certain depth without twist randomly on the left or right hand for each subject. During the tactile control stage, each subject was asked to maintain awake and rest for 5 min before the acupuncture twist starts by the acupuncturist. (III) Acupuncture manipulation: the lifting thrusting manipulation was performed for 2 min at the Hegu acupoint with the acupuncture needle twist frequency of 2 Hz. (IV) Post-manipulation tactile control: after the acupuncture manipulation period, each subject was asked to maintain awake and rest for 5 min. During this post-twist tactile control stage, the needle was kept at the Hegu acupoint without the twist. (V) Post-acupuncture rest control: after the acupuncturist removed the needle, each subject was asked to maintain awake and rest for 5 min without movement.

Immediately after the acupuncture experiment, the VAS *deqi* sensation behavior questionnaire was given to each subject to evaluate his/her *deqi* sensation for the following three stages: pre-manipulation tactile control, acupuncture manipulation, and post-manipulation tactile control (Supplementary Table S2).

### The Deqi Behavioral Index Calculation and Statistical Analysis

To comprehensively characterize the needing sensations, the *deqi* index was used for quantifying the *deqi* effect. The *deqi* behavioral index was defined as the summation of all the six individual needing sensation value according to the following equation (Supplementary Table S2):

(1) $$d\,e\,q\,i\,i\,n\,d\,e\,x=\sum_{i=1}^{N=6}S_{i}$$

where *S_i* indicates the rating points for the ith sensation and *N* represents the total number of different *deqi* sensations.

To evaluate if there exists significant *deqi* sensation difference between different conditions, the two-sample *t*-test was used on all subjects’ *deqi* indexes in MATLAB version R2016a for the following three stages: pre-manipulation tactile control, acupuncture manipulation, and post-manipulation tactile control. The *p*-Values were corrected with Bonferroni correction for multiple comparisons.

### Electroencephalography Recordings

The EEG signals were recorded by a Neuroscan SynAmps2 amplifier with 64 channels in the international 10–20 system. The reference electrode (REF) was placed on the right earlobe, and the ground electrode (GND) was placed on the forehead. The sampling frequency of the EEG signal was 1,000 Hz. For removing power line noise during the EEG data acquisition, the 50-Hz hardware notch filter was used.

### Electroencephalography Data Preprocessing

All the EEG data were preprocessed by using the EEGLAB toolbox (biosig extension) in MATLAB R2016a environment (The MathWorks, Natick, MA, United States). The data were first 0.5–60 Hz bandpass filtered with a 6,600th-order finite impulse response (FIR) filter and then were filtered by a 50-Hz notch filter to remove the power line noise (Kikuchi et al., 2011; Pedroni et al., 2017). Then, the blind source separation (BSS) algorithm was used to remove the interference of eye movements, electromyography (EMG), and other artifacts from the EEG data affected by artifacts (Gao et al., 2018). The BSS is based on second-order blind identification (SOBI)/canonical correlation analysis (CCA) to automatically remove electrooculogram (EOG)/EMG as described by previous studies (De Clercq et al., 2005; Gomez-Herrero et al., 2006). The EEG data were then re-referenced to the average reference and were bandpass filtered between 2 and 20 Hz with a 1,650th-order FIR filter (Koenig et al., 2002). After filtering, the data were segmented into 2-s epochs, in which epochs with an amplitude of more than ±80 μV at any electrode were rejected (Koenig et al., 2002; Gao et al., 2018).

### Electroencephalography Microstate Analysis

The EEG microstate analysis was processed with the EEGLAB’s microstate toolbox^1^ in the MATLAB R2016a environment. Microstate analysis followed the procedures described in Koenig et al. (1999); Brodbeck et al. (2012).

#### Global Field Power Calculation

As the first step, the global field power (GFP) at each time point was calculated on the preprocessed EEG data, which describes the spatial standard deviation of the EEG signal across all electrodes. The time series of GFP reflects the total energy change in the EEG topographic map over time. The GFP peaks have the highest energy with the optimal topographic signal-to-noise ratio (Lehmann and Skrandies, 1980; Koenig et al., 2002), which can characterize momentary quasi-stable voltage topography (Skrandies, 1989; Koenig and Brandeis, 2016).

(2) $$G\,F\,P\,\left(t\right)=\sqrt{\frac{\sum_{i=1}^{N}V_{i}\,{\left(t\right)}^{2}}{N}}$$

(N = number of electrodes, V_*i*_(t) = measured voltage of electrode i at time t, and i = electrode i).

#### The k-Means Clustering of Electroencephalography Topographies

**FIGURE 2:**
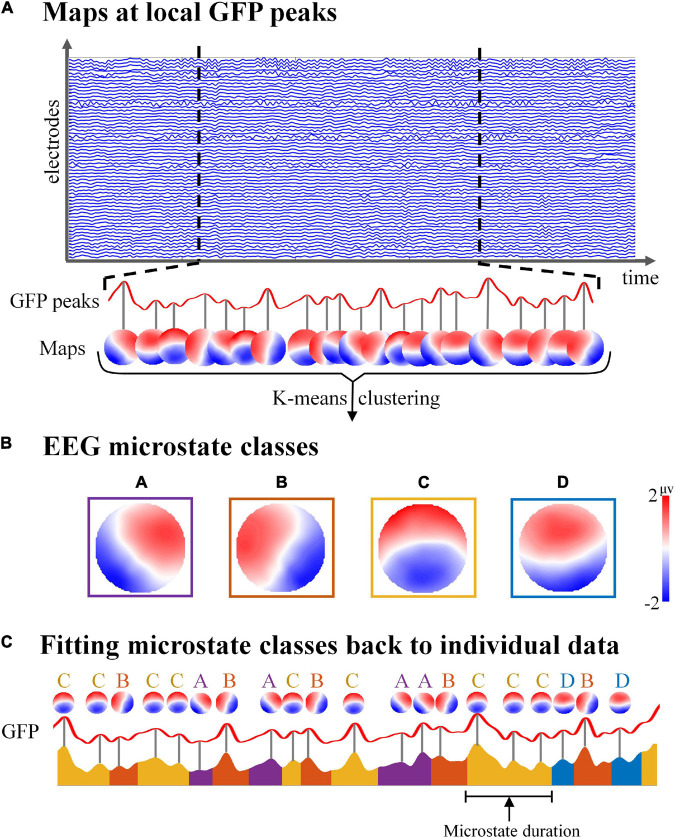
Schematic illustration of electroencephalography (EEG) microstate analysis. **(A)** The global field power (GFP) of spontaneous EEG was calculated, and all topographic maps at the maximum of local GFP were clustered by k-means algorithm. **(B)** Group template microstate maps according to the k-means clustering algorithm. **(C)** By computing the spatial correlation between the topographical at each GFP peak and each of the group microstate maps, the GFP peak was assigned to the microstate class with the highest correlation value. Then the microstate class sequence was obtained, which could be used to calculate the microstate parameters of each subject for each condition.

To get individual cluster maps, the k-means clustering analysis was first calculated on each individual subject for each condition separately. The GFP peaks were used as the initial topographic maps for clustering to maximize the topographic signal-to-noise ratio (Figure 2A). For each subject, the topographic map at GFP peaks was sent to the unsupervised k-means clustering algorithm (Koenig et al., 2002; Murray et al., 2008). The number of microstate classes was set to 4 in line with previous classical EEG microstate studies (Koenig et al., 2002; Brodbeck et al., 2012; Mishra et al., 2020), which could explain the most variance of map topographies during EEG microstate clustering (Koenig et al., 2002).

To identify the specific group maps for each condition, the individual cluster maps of all subjects were then averaged using a permutation algorithm. The algorithm determines the solution with the largest average correlation across all four classes of subjects, which is consistent with the previous study (Koenig et al., 1999; Milz et al., 2016). Finally, a specific set of group template microstate maps for each condition is obtained separately (Figure 2B).

#### Spatial Correlation of Electroencephalography Map Topographies and Sequence

To obtain the class label of each topography map at the GFP peak of original data and the corresponding class label sequence, the group template microstate maps were then fit back to each subject’s topographic maps at the GFP peaks by computing the spatial correlation between topographies (Figure 2C). To calculate the spatial correlation between the EEG microstate maps in our study with the corresponding classical microstate map templates (Koenig et al., 2002), the spatial correlation analysis method was applied in this study, which was consistent with the previous study (Brodbeck et al., 2012). The spatial correlation used the spatial Pearson’s product moment correlation coefficient (Brandeis et al., 1992), which was computed as follows:

(3) $$C=\frac{\sum_{i=1}^{N}\left(u_{i}\cdot v_{i}\right)}{\sqrt{\sum_{i=1}^{N}u_{i}^{2}}\cdot \sqrt{\sum_{i=1}^{N}v_{i}^{2}}}$$

(C = spatial correlation, N = number of electrodes, u = measured voltage map u, v = measured voltage map v, and i = electrode i).

The GFP peak was assigned to the microstate class with the highest spatial correlation value, and the middle time point between the two GFP peaks was set as the start and end points of each microstate (Koenig et al., 1999; Murray et al., 2008). In addition, the microstates at the beginning and end of each epoch were likely to be incomplete and were excluded from further analysis (Koenig et al., 2002).

Finally, the microstate time series of all subjects in each condition could be obtained through the microstate class label of each GFP peak in the original data (Figure 2C).

#### Electroencephalography Microstate Parameters

According to the calculated microstate time series, the following four microstate parameters can be calculated for each condition separately.

- Duration: the average time length during which each microstate remains stable. The microstate duration parameter is considered to reflect the average time for a set of neural generators to maintain synchronized activities (Lehmann et al., 1987; Strelets et al., 2003).
- Occurrence: during a given period, the average number of occurrences per second for each microstate. The microstate occurrence parameter represents the tendency of a set of neural generators to coordinate their activities over time (Lehmann et al., 1987; Khanna et al., 2014; Michel and Koenig, 2018).
- Contribution (time coverage): the proportion of each microstate’s occurrence time over the total time. The microstate contribution parameter is interpreted as reflecting the time coverage of its underlying neural generator relative to other neural generators (Lehmann et al., 1987). In addition, for the three parameters duration, occurrence, and contribution, knowing any two of them could calculate the remaining parameter (Lehmann et al., 2005).
- Transition probability (“microstate syntax”): percentage of transitions from one microstate class to another over all transitions occurring during a given period. The microstate transition probability is usually interpreted as different activation sequences of neural components that generate microstates (Lehmann et al., 2005; Khanna et al., 2015). If the transition from the previous microstate class to the next microstate class occurred randomly, then the relative occurrence of the microstate class would be proportional to the transition value (Lehmann et al., 2005).

### Statistical Analysis

#### Statistical Analysis of Microstate Parameters (Duration, Occurrence, and Contribution)

For each of the three microstate parameters (duration, occurrence, and contribution), separate 5 × 4 two-way repeated-measures analysis of variance (rmANOVA) was conducted using condition (pre-acupuncture rest control, pre-manipulation tactile control, acupuncture manipulation, post-manipulation tactile control, or post-acupuncture rest control) and microstate class (A, B, C, or D) as repeated measures (Table 1) in SPSS (Statistical Package for the Social Sciences v26, IBM). Mauchly’s test of sphericity was conducted during rmANOVA, and the Greenhouse–Geisser correction was applied when the sphericity assumption was not valid. Then, the *post hoc* paired *t*-tests were employed when the main effects or interactions in the rmANOVA were significant. To minimize the risk of type I errors, the *p*-Values were corrected with Bonferroni correction for multiple comparisons (Tomescu et al., 2014; Faber et al., 2017; Fu et al., 2018).

**TABLE 1:** Statistical description of microstate parameters and *post hoc* test results (two-way rmANOVA).

	Rest control (pre)		Tactile control (pre)		Acupuncture manipulation		Tactile control (post)		Rest control (post)		*Post hoc* comparisons—*p*-values (acupuncture manipulation vs. four controls) ^a^			
	Mean	(SEM)	Mean	(SEM)	Mean	(SEM)	Mean	(SEM)	Mean	(SEM)	Rest control (pre)	Tactile control (pre)	Tactile control (post)	Rest control (post)
**Dur (ms)**														

A	67.34	2.58	67.04	1.73	62.4	1.45	62.34	2.06	64.02	1.94	0.271	0.007*	1.000	1.000
B	64.74	3.29	62.88	2.98	62.0	2.61	62.73	2.38	65.27	2.76	0.807	1.000	1.000	0.908
C	61.36	2.60	61.12	3.23	74.6	4.83	57.19	1.52	57.94	1.78	0.008*	0.010*	0.011*	0.004**
D	70.46	4.79	74.13	4.58	59.5	1.71	80.03	6.39	73.85	3.89	*0*.*224*	*0*.*058*	*0*.*086*	0.016*

**Occ (/s)**														

A	4.02	0.15	4.10	0.20	3.93	0.16	3.84	0.21	3.90	0.18	1.000	1.000	1.000	1.000
B	3.73	0.18	3.74	0.21	3.64	0.22	3.95	0.20	3.96	0.18	1.000	1.000	0.490	0.618
C	3.49	0.27	3.28	0.26	4.39	0.16	3.23	0.25	3.37	0.25	0.032*	0.006*	0.004**	0.008*
D	4.06	0.22	4.23	0.16	3.57	0.24	4.27	0.13	4.20	0.19	0.030*	*0*.*172*	0.049*	0.017*

**Con (%)**														

A	26.66	1.32	27.11	1.52	24.26	1.07	23.45	1.58	24.60	1.46	0.338	0.091	1.000	1.000
B	23.95	1.74	23.27	1.75	22.34	1.69	24.51	1.56	55.59	1.56	1.000	1.000	0.586	0.129
C	21.38	1.77	29.80	1.84	32.03	2.30	18.61	1.73	19.46	1.66	0.003**	0.002**	0.003**	0.001**
D	28.02	2.44	29.63	2.12	21.36	1.83	33.43	2.75	30.36	2.13	0.012*	0.030*	0.026*	0.005*

Significant differences are displayed in bold type, and trends are shown in italics. Rest control (pre), pre-acupuncture rest control; Tactile control (pre), pre-manipulation tactile control; Tactile control (post), post-manipulation tactile control; Rest control (post), post-acupuncture rest control. Dur, duration; Occ, occurrence; Con, contribution. ^*a*^Adjustment for multiple comparisons: Bonferroni. *p < 0.05 and **p < 0.005.

#### Statistical Analysis of Microstate Parameter (Transition Probability)

For each of the 12 transition probability parameters (A→B, A→C, A→D, B→A, B→C, B→D, C→A, C→B, C→D, D→A, D→B, and D→C), separate one-way five-level rmANOVA was conducted using condition (pre-acupuncture rest control, pre-manipulation tactile control, acupuncture manipulation, post-manipulation tactile control, or post-acupuncture rest control) as repeated measures (Table 2). Mauchly’s test of sphericity was conducted during rmANOVA, and the Greenhouse–Geisser correction was applied when the sphericity assumption was not valid. The above statistical analysis was computed using SPSS (Statistical Package for the Social Sciences v26, IBM).

**TABLE 2:** Percentage of transitions in microstate class.

Transition (%)	Rest control (pre)		Tactile control (pre)		Acupuncture manipulation		Tactile control (post)		Rest control (post)	
	Mean	SEM	Mean	SEM	Mean	SEM	Mean	SEM	Mean	SEM
A to B	8.74	0.69	8.79	0.87	7.88	0.73	8.79	0.90	8.92	0.80
A to C	7.58	0.69	7.59	0.66	10.30	0.77	6.14	0.59	6.37	0.63
A to D	9.92	0.79	10.76	0.91	6.96	0.60	9.99	0.64	9.86	0.75
B to A	8.86	0.60	8.82	0.83	7.75	0.70	8.80	0.85	9.05	0.82
B to C	7.05	0.77	6.05	0.54	9.17	0.63	6.40	0.48	6.90	0.51
B to D	8.47	0.71	8.74	0.68	6.34	0.60	10.39	0.66	9.67	0.76
C to A	7.50	0.70	7.76	0.75	10.43	0.74	6.15	0.63	6.61	0.65
C to B	6.88	0.68	5.51	0.55	9.11	0.56	6.19	0.54	6.88	0.53
C to D	8.30	0.66	8.20	0.73	9.35	0.85	8.44	0.79	8.07	0.80
D to A	9.93	0.71	10.40	0.87	6.89	0.62	9.98	0.66	9.47	0.63
D to B	8.75	0.65	9.46	0.74	6.44	0.54	10.59	0.67	9.91	0.77
D to C	8.01	0.72	7.93	0.67	9.40	0.75	8.13	0.74	8.43	0.69
Total	100	–	100	–	100	–	100	–	100	–

*Rest control (pre), pre-acupuncture rest control; Tactile control (pre), pre-manipulation tactile control; Tactile control (post), post-manipulation tactile control; Rest control (post), post-acupuncture rest control.*

To further compare the transition probability parameters for different conditions, the two-sample *t*-tests were performed on the transition probabilities between acupuncture manipulation and all controls, and those between tactile controls and rest controls in MATLAB version R2016a, separately. To minimize the risk of type I errors, the *p*-Values were corrected with Bonferroni correction for multiple comparisons.

#### Correlation Analysis Between Neural Responses and Acupuncture Behavior Scores

To further investigate if the neural responses are significantly correlated with acupuncture’s behavior scores, Pearson’s correlation and linear regression analysis were conducted between *deqi* behavior data and EEG microstate parameters (duration, occurrence, contribution, and transition probability) for acupuncture manipulation, and pre- and post-manipulation tactile controls, separately. To keep the sample size consistent during Pearson’s correlation calculating under different conditions, the *x*-axis (behavior) and *y*-axis (microstate parameters) values of tactile controls’ each data sample were obtained by averaging the corresponding *x*-axis and *y*-axis data of the pre- and post-manipulation tactile controls, respectively. During the linear regression analysis, the 95% confidence intervals for the mean of the polynomial evaluation were also computed.

## Results

### Behavior *Deqi* Results for Different Conditions

**FIGURE 3:**
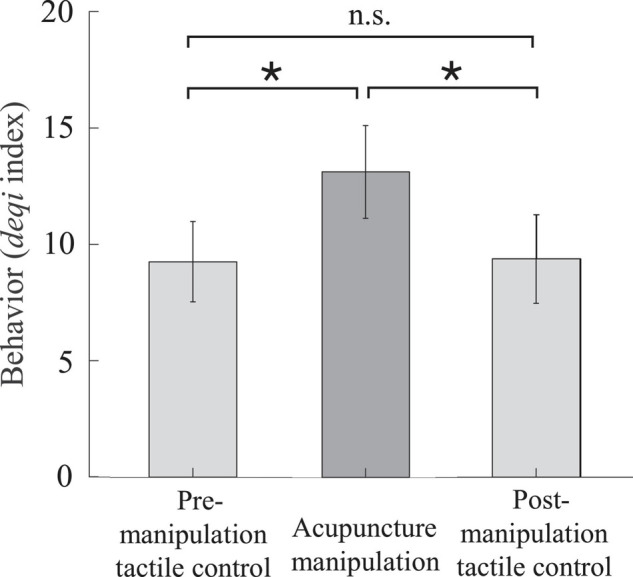
The acupuncture behavior scores quantified by the *deqi* index were significantly higher for acupuncture manipulation compared with tactile controls. Acupuncture manipulation resulted in a significantly increased *deqi* index compared with pre-manipulation tactile control and post-manipulation tactile control (mean ± SEM, paired *t*-test, Bonferroni correction for multiple comparisons, *p < 0.05, *n* = 16; the number of multiple comparisons = 3).

To validate the behavior effectiveness of acupuncture manipulation, we compared the *deqi* behavior scores of the acupuncture manipulation with those of the pre- and post-manipulation tactile controls (Figure 3 and Supplementary Table S2). The subjects’ *deqi* indexes of the acupuncture manipulation were significantly larger than those of pre-manipulation tactile control (*p* = 0.036, paired *t*-test, Bonferroni correction) and were significantly larger than those of post-manipulation tactile control (*p* = 0.037, paired *t*-test, Bonferroni correction). There was no significant difference between pre-manipulation tactile control and post-manipulation tactile control. These results indicated that acupuncture manipulation condition could significantly cause behavioral effects compared with pre- and post-manipulation tactile control conditions.

### The Electroencephalography Microstate Results Were Consistent With Previous Classical Findings

**FIGURE 4:**
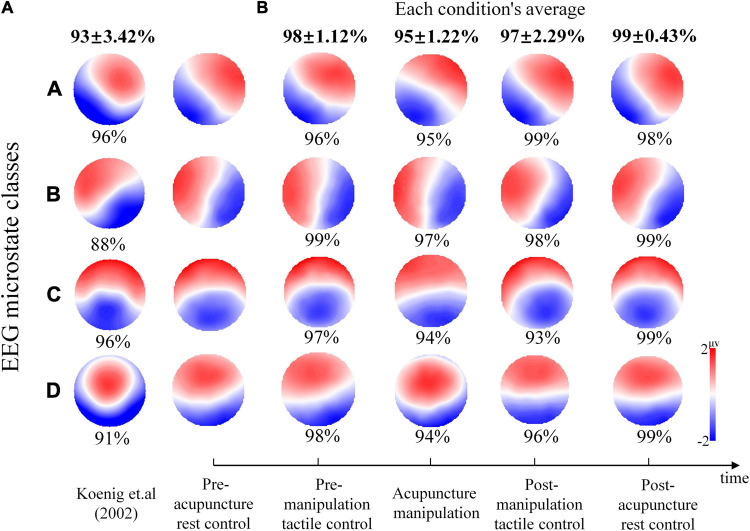
The spatial correlation percent and electroencephalography (EEG) microstate maps during different conditions. **(A)** The percent spatial correlation of this study’s pre-acupuncture rest control microstate topography maps with the rest of the maps of previous publications (Koenig et al., 2002). The percent spatial correlation is given below each microstate class map. **(B)** The EEG microstate maps during different conditions. The percent spatial correlation between pre-acupuncture rest control maps with the other four conditions maps is given below each map. The average correlation for each condition is given at the top of each column with bold type. Colors represent the relative potential distribution.

To investigate whether this study’s EEG microstate maps are consistent with those of previous results, microstate map comparisons and the spatial correlations were analyzed. The microstate analysis was performed on the entire data of all five conditions, and the corresponding microstate maps were obtained (Figure 4), in which the four microstate maps were marked as A, B, C, and D according to the maximum spatial correlation value with the previous classical microstate maps (Koenig et al., 2002). Microstate map A showed a left occipital to right frontal orientation, and microstate B showed a right occipital to left frontal orientation. Microstate C showed an approximately symmetric occipital to frontal orientation, and microstate D was characterized by a frontal–central maximum. The characteristics of this study’s four microstate maps were in line with previous findings (Koenig et al., 2002; Britz et al., 2010). Furthermore, the spatial correlations on rest condition’s microstate maps between this study and previous publication (Figure 4A) were analyzed to investigate whether our microstate results were consistent with previous classical results (Koenig et al., 2002). It was showed that our four microstate maps of the pre-acupuncture rest control had relatively high spatial correlation values (mean ± SD, 93% ± 3.42%; A = 96%, B = 88%, C = 96%, and D = 91%) with maps described in Koenig et al. (2002) (Figure 4A).

### Correlation Results Between Microstate Maps of Different Conditions

To investigate the microstate map changes during different conditions, spatial correlations of map topographies between the pre-acupuncture rest control and the next four conditions were analyzed for each microstate class separately. It was shown that compared with the pre-acupuncture rest control’s microstate maps, the other four conditions’ microstate maps overall showed relatively high spatial correlation (the pre-manipulation tactile control, mean ± SD, 98% ± 1.12%; the post-manipulation tactile control, mean ± SD, 97% ± 2.29%; acupuncture manipulation, mean ± SD, 95% ± 1.22%; and post-acupuncture rest control, mean ± SD, 99% ± 0.43%) (Figure 4B). Interestingly, the acupuncture manipulation’s four class maps showed the lowest concordance with the pre-acupuncture rest control maps (mean ± SD, 95% ± 1.22%), in which the spatial correlation percent for microstates C and D was the smallest among all results (C = 94% and D = 94%) (Figure 4B). On the other hand, the post-acupuncture rest control maps showed the overall highest concordance with the pre-acupuncture rest control’s microstate maps (mean ± SD, 99% ± 0.43%). These phenomena showed that microstate maps were first modulated by acupuncture manipulation and then recovered back to the baseline resting state. All these results indicated that the EEG microstate maps could be a promising objective marker to reveal the acupuncture effects.

### Acupuncture Manipulation Increased the Duration, Occurrence, and Contribution Parameters of Microstate C, Whereas It Decreased Those of Microstate D

**FIGURE 5:**
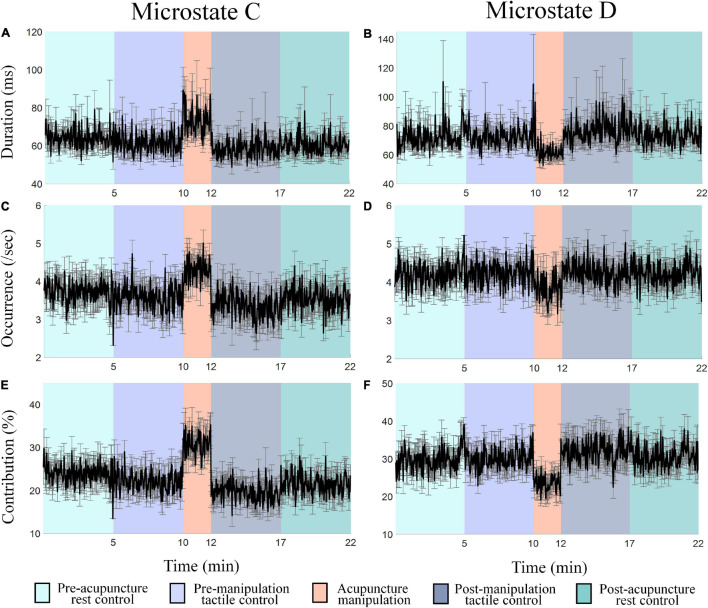
**(A–F)** The time series of microstates C’s and D’s parameters (duration, occurrence, and contribution) changes over time in different conditions. Each data point is the mean value of all subjects within 2 s. Colors represent different conditions, and the error bar represents mean ± SEM.

To further investigate the spatial correlation changes for microstates C and D, the time series of microstate C’s and D’s parameters (duration, occurrence, and contribution) changes over time in different conditions were obtained (Figure 5). Results showed different parameters’ change patterns for microstates C and D under acupuncture manipulation. Microstate C’s duration, occurrence, and contribution parameters had tremendous increases during acupuncture manipulation than the other four controls (Figures 5A,C,E). On the contrary, microstate D’s duration, occurrence, and contribution parameters had obvious decreases during acupuncture manipulation as compared with other controls (Figures 5B,D,F). These results indicated that the microstate dynamics parameters could reveal the acupuncture’s neural modulation effects on the human brain.

### Significant Condition × Microstate Class Interaction Effect for Duration, Occurrence, and Contribution Parameters

**FIGURE 6:**
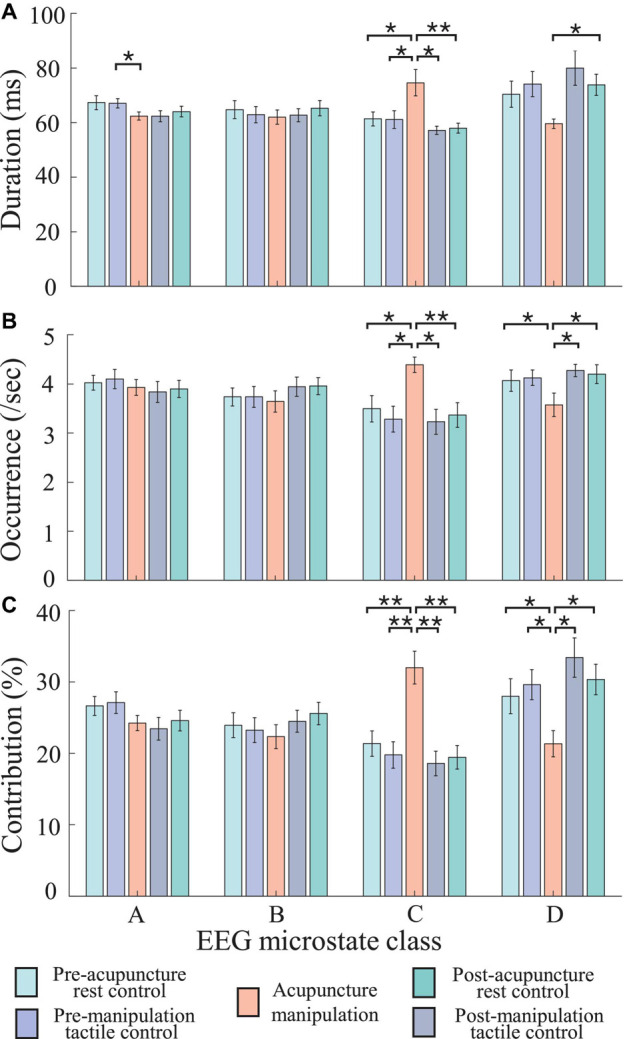
Statistical comparisons of microstate parameters (duration, occurrence, and contribution) between different conditions (pre-acupuncture rest control, pre-manipulation tactile control, acupuncture manipulation, post-manipulation tactile control, and post-acupuncture rest control) in different microstate classes (A-D). **(A)** Duration. **(B)** Occurrence. **(C)** Contribution. Colors represent different conditions, and error bar represents mean ± SEM (5 × 4 two-way repeated-measures ANOVA (rmANOVA), *post hoc* paired *t*-tests with Bonferroni correction for multiple comparisons, *p < 0.05 and **p < 0.005, *n* = 16; the number of multiple comparisons = 40).

To further quantify the microstate parameter changes between different conditions (pre-acupuncture rest control, pre-manipulation tactile control, acupuncture manipulation, post-manipulation tactile control, and post-acupuncture rest control) in different microstate classes (A, B, C, and D), the two-way rmANOVA was conducted for duration, occurrence, and contribution parameter, separately (Figure 6 and Table 1). To keep each condition’s data balance and also to avoid the non-stationarity during conditions transition, each condition’s intermediate 1-min data were selected. Overall, the two-way rmANOVA showed significant condition × microstate class interaction effect for duration, occurrence, and contribution microstate parameters: duration [*F*_(df1 = 2.98, df2 = 44.72, ϵ = 0.248)_ = 7.92, *p* = 2.14 × 10^–4^], occurrence [*F*_(__*df1* = 5.01, df2 = 75.15, ϵ = 0.417)_ = 8.65, *p* = 2 × 10^–6^], and contribution [*F*_(__*df1* = 2.73, df2 = 40.92, ϵ = 0.227)_ = 11.03, *p* = 3.2 × 10^–5^], respectively. And there was no significant main effect.

### The Duration, Occurrence, and Contribution Parameters of Microstate C Were Significantly Increased by Acupuncture Manipulation

The *post hoc* rmANOVA results showed that microstate C’s parameters (duration, occurrence, and contribution) of acupuncture manipulation were significantly larger than those of other conditions (Figure 6 and Table 1). First, microstate C’s duration of acupuncture manipulation was significantly larger than that of the other conditions (pre-acupuncture rest control, *p* = 0.008^∗^; pre-manipulation tactile control, *p* = 0.010^∗^; post-manipulation tactile control, *p* = 0.011^∗^; post-acupuncture rest control, *p* = 0.004^∗∗^; alpha = 0.05; Bonferroni correction) (Figure 6A and Table 1). Second, microstate C’s occurrence of acupuncture manipulation was also significantly higher than that of the other conditions (pre-acupuncture rest control, *p* = 0.032^∗^; pre-manipulation tactile control, *p* = 0.006^∗^; post-manipulation tactile control, *p* = 0.004^∗∗^; post-acupuncture rest control, *p* = 0.008^∗^; with Bonferroni correction) (Figure 6B and Table 1). Finally, microstate C’s contribution of acupuncture manipulation was significantly higher than that of the other conditions (pre-acupuncture rest control, *p* = 0.003^∗∗^; pre-manipulation tactile control, *p* = 0.002^∗∗^; post-manipulation tactile control, *p* = 0.003^∗∗^; post-acupuncture rest control, *p* = 0.001^∗∗^; with Bonferroni correction) (Figure 6C and Table 1). These changes were consistent with the previous time series results of microstate C’s parameters (duration, occurrence, and contribution) increasing changes during acupuncture manipulation (Figure 5).

### The Duration, Occurrence, and Contribution Parameters of Microstate D Were Significantly Decreased by Acupuncture Manipulation

On the contrary, the *post hoc* rmANOVA results showed that microstate D’s parameters (duration, occurrence, and contribution) of acupuncture manipulation were significantly smaller than those of other conditions (Figure 6 and Table 1). First, microstate D’s duration of acupuncture manipulation was smaller than that of other conditions and was significantly lower than that of the post-acupuncture rest control (*p* = 0.016^∗^; with Bonferroni correction) (Figure 6A and Table 1). Second, microstate D’s occurrence of acupuncture manipulation was lower than that of other conditions and was significantly lower than that of the other conditions (pre-acupuncture rest control, *p* = 0.030^∗^; post-manipulation tactile control, *p* = 0.049^∗^; post-acupuncture rest control, *p* = 0.017^∗^; with Bonferroni correction) (Figure 6B and Table 1). Finally, microstate D’s contribution of acupuncture manipulation was significantly lower than that of the other conditions (pre-acupuncture rest control, *p* = 0.012^∗^; pre-manipulation tactile control, *p* = 0.030^∗^; post-manipulation tactile control, *p* = 0.026^∗^; post-acupuncture rest control, *p* = 0.005^∗^; with Bonferroni correction) (Figure 6C and Table 1). These changes were consistent with the previous time series results of microstate D’s parameters (duration, occurrence, and contribution) decreasing changes during acupuncture manipulation (Figure 5).

### The Duration Parameter of Microstate C Was Significantly Positively Correlated With the Behavior Score During Acupuncture Manipulation

**FIGURE 7:**
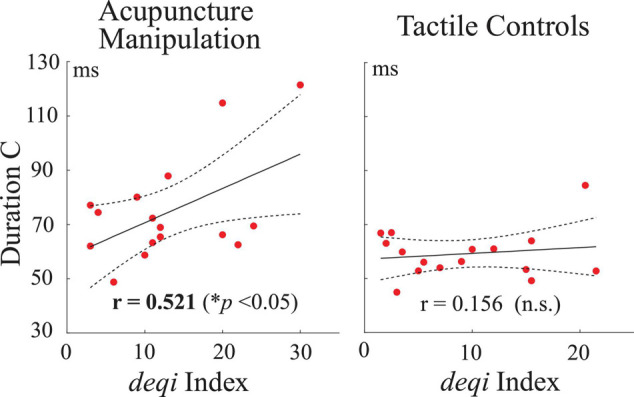
Correlation analysis between neural responses and acupuncture’s behavior scores across all subjects. Pearson’s correlations between the duration parameter of microstate C and the *deqi* behavior index for the acupuncture manipulation and tactile controls. Each red dot is indicated for each subject’s data. Dashed lines represent the 95% confidence intervals for the mean of polynomial evaluation. Solid lines represent linear regression (*p* < 0.05, *n* = 16).

To further investigate if there existed relationships between neural responses (parameters of EEG microstates C and D) and acupuncture behavior scores, Pearson’s correlations were performed between the microstate parameters (duration, occurrence, and contribution) and each subject’s behavior *deqi* index. Interestingly, the duration parameter of microstate C was found to have a significant positive correlation with acupuncture’s behavior *deqi* index during the acupuncture manipulation (*r* = 0.521, *p* = 0.038), whereas there was no significant correlation for the tactile controls (*r* = 0.156, n.s.) (Figure 7). Besides, the contribution parameter of microstate C had a positive correlation tendency (*r* = 0.433, n.s.) with behavior *deqi* index during acupuncture manipulation compared with tactile controls (*r* = −0.136, n.s.) (Supplementary Figure S1). Besides, no significant correlation was found for any other conditions. The neural behavior correlation results indicated that the duration parameter of microstate C could be an effective marker to reflect subjects’ acupuncture *deqi* behavior scores during acupuncture manipulation.

### Acupuncture Manipulation Significantly Increased Transition Probabilities With Microstate C as the Node, Whereas It Decreased Those With Microstate D as the Node

**FIGURE 8:**
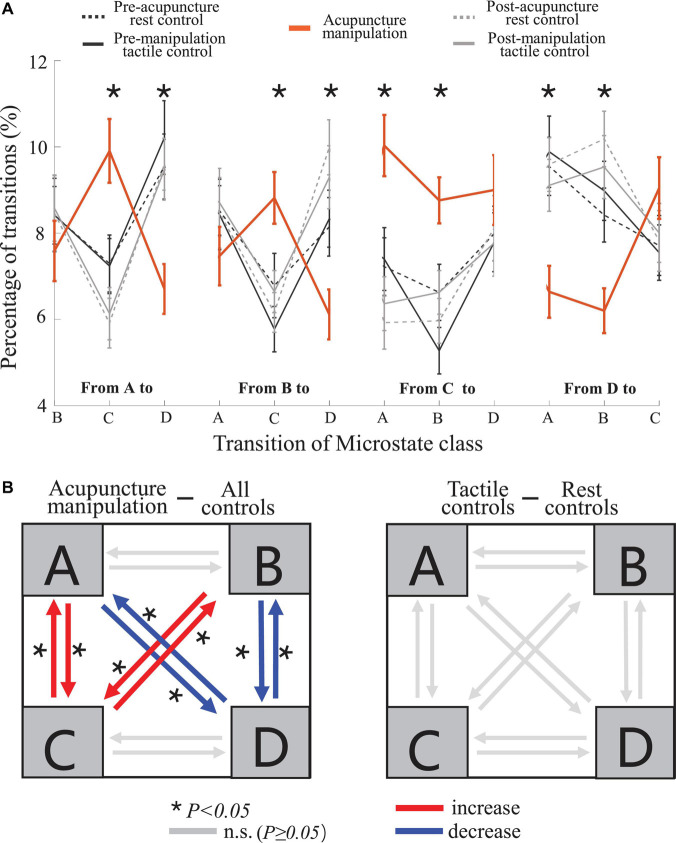
Transition probability trend and statistical comparisons for different conditions. **(A)** Trends of the transition probability for the acupuncture manipulation and the remaining four controls, in which the lines represent the switching probability from a microstate class to another. For each of the 12 transition probability parameters, separate one-way five-level repeated-measures analysis of variance (rmANOVA) was conducted using condition as repeated measures (mean ± SEM, *p < 0.05, *n* = 16). **(B)** Schematic arrangement and statistical comparisons of the transition probability. Left: comparison between acupuncture manipulation (*n* = 16) and all controls (combining four controls, *n* = 64). Right: comparison between tactile controls (combining two tactile controls, *n* = 32) and rest controls (combining two rest controls, *n* = 32). Two-sample *t*-tests with Bonferroni correction for multiple comparisons, *p < 0.05; the number of multiple comparisons = 12.

To further reveal the temporal dynamics during acupuncture, the transition probability between different microstate classes were compared between different conditions (Figure 8 and Supplementary Figure S2). Interestingly, the other four controls showed similar changing trend in the transition probability, whereas the acupuncture manipulation showed a unique and almost opposite changing trend (Figure 8A). Furthermore, the separate one-way rmANOVA showed significant difference between the five conditions for the transition probabilities with microstate C as the node [A→C, *F*_(__*df1* = 2.17, df2 = 31.74, ϵ = 0.529)_ = 11.62, *p* = 1.3 × 10^–4^; B→C, *F*_(__*df1* = 4, df2 = 12)_ = 6.375, *p* = 0.005; C→A, *F*_(__*df1* = 1.91, df2 = 28.58, ϵ = 0.476)_ = 12.11, *p* = 1.9 × 10^–4^; and C→B, *F*_(__*df1* = 4, df2 = 12)_ = 7.45, *p* = 0.003], and for the transition probabilities with microstate D as the node [A→D, *F*_(__*df1* = 2.01, df2 = 30.18, ϵ = 0.502)_ = 9.16, *p* = 0.001; B→D, *F*_(__*df1* = 4, df2 = 12)_ = 4.80, *p* = 0.015; D→A, *F*_(__*df1* = 2.02, df2 = 30.35, ϵ = 0.506)_ = 10.76, *p* = 2.8 × 10^–4^; and D→B, *F*_(__*df1* = 4, df2 = 12)_ = 5.83, *p* = 0.008] (Figure 8A). Microstate C as the node means transition probabilities to or from microstate C; and microstate D as the node means transition probabilities to or from microstate D.

Interestingly, the acupuncture manipulation showed a significant increase for the transition probabilities with microstate C as the node [A→C, *t*(78) = 4.49, *p* = 3.0 × 10^–4^; B→C, *t*(78) = 3.89, *p* = 0.003; C→A, *t*(78) = 4.40, *p* = 4.2 × 10^–4^; and C→B, *t*(78) = 4.26, *p* = 6.7 × 10^–4^; two-sample *t*-tests with Bonferroni correction], compared with all controls (combining four controls) (Figure 8B and Table 2). On the other hand, the acupuncture manipulation showed a significant decrease for the transition probabilities with microstate D as the node [A→D, *t*(78) = −3.86, *p* = 0.003; B→D, *t*(78) = −3.85, *p* = 0.003; D→A, *t*(78) = −3.96, *p* = 0.002; and D→B, *t*(78) = −4.26, *p* = 6.8 × 10^–4^; two-sample *t*-tests with Bonferroni correction], compared with all controls (combining four controls) (Figure 8B and Table 2). For the control group, there was no significant difference for the transition probability between tactile controls and rest controls (Figure 8B). In addition, compared with rest controls (combining two rest controls) or with tactile controls (combining two tactile controls), the acupuncture manipulation also showed significant increase for the transition probabilities with microstate C as the node, but significant decrease for the transition probabilities with microstate D as the node (Supplementary Figure S2).

### The Parameter of Transition Probability B→C Was Significantly Positively Correlated With the Behavior Score During Acupuncture Manipulation

**FIGURE 9:**
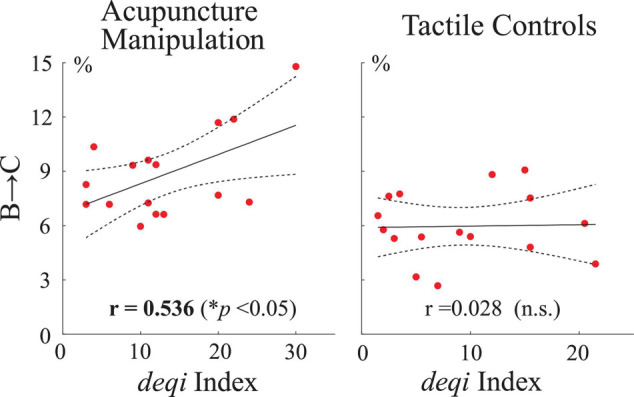
Correlation analysis between neural responses and acupuncture’s behavior scores across all subjects. Pearson’s correlations between the transition probability parameter B→C and the *deqi* behavior index for the acupuncture manipulation and tactile controls, respectively. Each red dot is indicated for each subject’s data. Dashed lines indicate the 95% confidence intervals for the mean of polynomial evaluation. Solid lines represent linear regression. *p < 0.05, *n* = 16.

To further investigate if there existed relationships between neural responses (parameters of transition probability with microstates C and D as nodes) and acupuncture behavior scores, Pearson’s correlations were performed between the microstate’s transition probability parameters and each subject’s behavior *deqi* index. Interestingly, the transition probability from microstate B to C was found to have a significant positive correlation with acupuncture’s behavior *deqi* index during the acupuncture manipulation (*r* = 0.536, *p* = 0.032), whereas there was no significant correlation for the tactile controls (*r* = 0.028, n.s.) (Figure 9). Besides, no significant correlation was found for any other conditions. These results further indicated that B→C transition probability parameters could be an effective marker to reflect subjects’ acupuncture *deqi* behavior scores during acupuncture manipulation.

## Discussion

### Electroencephalography Microstates Reveal the Neuromodulation Effects of Acupuncture With Deqi

Previous studies have stated that microstates are atoms of thought, which can represent basic cognitive processes in different states and tasks (Koenig et al., 2002; Brodbeck et al., 2012; Milz et al., 2016). To the best of our knowledge, this study was the first to propose the idea of EEG microstates to study the neuromodulation mechanism of acupuncture. And our results showed that EEG microstates could be used as novel markers to reveal the neuromodulation effects of acupuncture with *deqi*. First, microstate C was significantly increased during acupuncture stimulation (Figures 5, 6). On the other hand, microstate D was significantly decreased during acupuncture stimulation (Figures 5, 6). Second, the transition probabilities with microstate C as nodes were significantly increased (Figure 8 and Supplementary Figure S2), whereas the transition probabilities with microstate D as nodes were significantly decreased during acupuncture stimulation (Figure 8 and Supplementary Figure S2). Moreover, the microstate parameters (microstate C’s duration and microstate B→C’s transition probability) showed a significantly positive correlation with acupuncture *deqi* behavior scores (Figures 7, 9). Taken together, the EEG microstates C and D could provide novel neuroimaging evidence for revealing the neuromodulation effects of acupuncture with *deqi*.

### Acupuncture Manipulation Could Cause Significantly Increased Deqi Behavior Effects

The acupuncture *deqi* sensation that patients experienced could be a valuable behavioral index to reflect the acupuncture effect according to the TCM (Hui et al., 2007; Yu et al., 2012). Behavior results showed that the subjects’ *deqi* indexes of the acupuncture manipulation were significantly larger than those of pre-/post-manipulation tactile control conditions. And there was no significant difference between pre-manipulation and post-manipulation tactile controls (Figure 3). These results indicate that acupuncture manipulation could effectively cause *deqi* behavioral effects as compared with control conditions. Therefore, this experimental design is effective and usable.

### Electroencephalography Microstate Maps and the Corresponding Functional Networks

By comparing our results with previous classical EEG microstate maps, the observed four EEG microstate topography maps (A, B, C, and D) during acupuncture were in line with previous findings (Koenig et al., 2002; Figure 4A). Moreover, previous EEG-fMRI simultaneous recording studies showed that the four EEG microstate topography maps were related to different brain functional networks (Britz et al., 2010; Musso et al., 2010; Van de Ville et al., 2010). Microstates A and B were related to the auditory and visual networks, respectively (Britz et al., 2010); and microstates C and D were related to the salience network (Britz et al., 2010; Michel and Koenig, 2018) and DAN, respectively (Smith et al., 2009; Britz et al., 2010; Michel and Koenig, 2018). In this study, the four EEG microstate topography maps during acupuncture manipulation showed the most dramatic changes with the lowest spatial correlation with pre-acupuncture rest control (Figure 4B). We speculated that the corresponding functional networks could be modulated by acupuncture manipulation. Furthermore, microstates C and D had the smallest correlation values (Figure 4B), which may mean that the corresponding salience network and DAN were modulated by acupuncture manipulation. In addition, the post-acupuncture rest control maps recovered back to the baseline condition and showed the overall highest concordance with pre-acupuncture rest control (Figure 4B), which may indicate that the corresponding functional networks were modulated by acupuncture manipulation and then recovered back to the baseline resting state.

### Microstate C Could Be an Objective Marker for Acupuncture Manipulation

The acupuncture *deqi* sensation that patients experienced was a valuable behavioral index to reflect the acupuncture effect according to the TCM (Hui et al., 2007; Yu et al., 2012). However, the current behavioral evaluation of *deqi* is subjective, which needs an objective marker. Here, first, the EEG microstate C’s parameters (duration, occurrence, and contribution) showed the most obvious and significant increase during acupuncture manipulation, compared with other controls (Figures 5, 6). This indicates that microstate C could be a potential marker for evaluate neuromodulation effects of acupuncture with *deqi*. Second, compared with tactile controls, the duration parameter of microstate C was significantly positively correlated with the acupuncture *deqi* behavior index during acupuncture manipulation (Figure 7). It meant that microstate C’s duration parameter could reflect subject’s *deqi* behavior index. Moreover, the previous study showed that the EEG microstate C had significant changes for adolescents with 22q11 deletion syndrome compared with normal control, indicating that microstate C could be a marker for the early diagnosis of schizophrenia (Tomescu et al., 2014). Therefore, we speculated that our findings also prove that the EEG microstate C could be an objective marker to evaluate neuromodulation effects of acupuncture with *deqi*.

### Acupuncture Modulated the Salience Network Corresponding to Microstate C

Previous EEG-fMRI simultaneous recording studies showed that the EEG microstate C was associated with the neural activities of brain regions including the right anterior insula, the anterior cingulate, and bilateral inferior brand gyri (Britz et al., 2010). Therefore, the observed microstate C’s increase indicated that these underlying brain regions were engaged during the acupuncture manipulation, which was consistent with the previous fMRI finding of the insula and anterior cingulate cortex’s activations during acupuncture (Fang et al., 2009; Chae et al., 2013). Furthermore, the anterior insula and anterior cingulate regions corresponding to microstate C belong to the salience network (Britz et al., 2010), which indicates that the salience network could be modulated during acupuncture stimulation.

In addition, it has been shown that the salience network could coordinate the neural resources to important self-related cognition and external stimuli of importance (Menon, 2011; Palaniyappan and Liddle, 2012; Uddin, 2015), by mediating the switch between the default mode network (DMN) and the central executive network (CEN) (Uddin, 2015). Therefore, it is reasonable to speculate that by activating the salience network, the external acupuncture stimulation helps to balance the activity of DMN and CEN and finally contributes to coordinate the self-related (internally directed) and goal-oriented (externally directed) cognition (Uddin, 2015). This helps to explain the previous finding that acupuncture at the Hegu acupoint could improve the cognitive ability of Alzheimer’s disease patients by modulating the connectivity of large-scale network (Liang et al., 2014).

### Acupuncture Modulated the Transition Probabilities With Microstate C as Node

Compared with those of controls, transition probabilities with microstate C as node were significantly increased during acupuncture manipulation (Figure 8). Especially, the transition probability of microstate B→C was significantly positively correlated with the *deqi* index during acupuncture manipulation (Figure 9). These results add more evidence for microstate C as an objective marker for acupuncture manipulation with *deqi*.

In addition, previous EEG-fMRI simultaneous study showed that EEG microstates could reveal the dynamic patterns of EEG activities (Lehmann et al., 1987; Khanna et al., 2014; Michel and Koenig, 2018), and especially transition probabilities could speculate the dynamic switching patterns of the underlying functional networks (Britz et al., 2010). And microstates A, B, and C were related with auditory network, visual network, and salience network, respectively, by previous EEG-fMRI study (Smith et al., 2009; Britz et al., 2010; Michel and Koenig, 2018). Therefore, we speculate that acupuncture manipulation could enhance the dynamic information exchanges between salience network and auditory/visual sensory networks, thus improving the ability for cognitive information processing (Brodbeck et al., 2012), which could be further evidenced by microstate C’s significantly increased transition probabilities with both microstates A and B (Figures 8A,B). This could further explain previous findings that acupuncture stimulation could strengthen brain connectives (Yu et al., 2017, 2018).

Furthermore, the salience network was found to mediate neural resources to self-related cognition and external stimuli of importance (Menon, 2011; Palaniyappan and Liddle, 2012; Uddin, 2015). Therefore, it is reasonable to speculate that by enhancing the connections between salience network and sensory network, acupuncture could help to coordinate the internally or externally directed cognition. Taken together, the significantly increased transition probabilities with microstate C as node not only add more evidence for microstate C as an objective marker but also help to reveal the exchanging information between different functional networks during acupuncture manipulation.

### Acupuncture Manipulation Modulated Microstate D and Its Corresponding Dorsal Attention Network

Compared with those of controls, the EEG microstate D’s duration, occurrence, and contribution parameters showed significant decrease during acupuncture manipulation (Figures 5, 6). Here, we argue that microstate D could also be a marker to evaluate neuromodulation effects of acupuncture with *deqi*. Moreover, previous EEG-fMRI simultaneous study showed that the EEG microstate D was related with right-lateralized frontal and parietal cortex (Britz et al., 2010). And the frontoparietal cortex corresponding to microstate D belongs to the DAN (Seitzman et al., 2017; Michel and Koenig, 2018), which is responsible for the cognitive selection of sensory stimuli and responses (Corbetta and Shulman, 2002; Fox et al., 2006; Kim, 2014). Therefore, we speculate that the DAN is inhibited during the acupuncture manipulation, which is evidenced by the decrease of microstate D. In addition, the Hegu acupoint is considered to have analgesic effects (White et al., 2003; Kong et al., 2005) and is commonly used in the treatment of painful syndrome (Andersson and Lundeberg, 1995). We further postulate that by inhibiting the DAN, acupuncture manipulation could decrease the top-down attention to perceive pain sensory information, which could explain the analgesic effects of acupuncture at the Hegu acupoint. Taken together, we argue that the EEG microstate D could also be an objective marker to evaluate neuromodulation effects of acupuncture with *deqi*.

### Acupuncture Modulated the Transition Probabilities With Microstate D as Node

Compared with those of controls, the transition probabilities with microstate D as node were significantly decreased during acupuncture manipulation (Figure 8), which adds more evidence for microstate D as an objective marker for acupuncture manipulation. In addition, previous EEG-fMRI simultaneous studies showed that microstate D was associated with DAN (Smith et al., 2009; Britz et al., 2010; Michel and Koenig, 2018). We further speculate that acupuncture could inhibit the information processing of DAN with the auditory and visual sensory network, which could be evidenced by microstate D’s decreased transition probabilities with both microstates A and B (Figures 8A,B). By inhibiting the connections of DAN with other sensory networks, acupuncture manipulation could help reduce the subject’s top-down attention to perceive sensory stimuli, which could further explain the analgesic effects of acupuncture (Hui et al., 2005; Chae et al., 2013). Taken together, the significantly decreased transition probabilities with microstate D as node not only add more evidence for microstate D as an objective marker but also help to reveal the exchanging information between different functional networks during acupuncture manipulation.

### Limitations and Future Works

In addition, there were several potential limitations in this study. First, more participations should be recruited to provide more evidence for further exploring the correlation relationship between neural responses with behavior scores in the following study. For example, although there existed statistical significance, the statistical power and significance of the correlation results between behavior scores with microstate C’s duration (Figure 7) and B→C’s transition probability (Figure 9) parameters could be further enhanced and validated by recruiting more participations. Second, the temporal dynamics modulation of functional networks during acupuncture revealed by current EEG microstate analysis should be further explored and validated using the fMRI-EEG simultaneous recording method. The fMRI-EEG simultaneous recording could add more evidence for explaining the underlying functional networks changes revealed by different EEG microstates during acupuncture. Third, this study only explored the effects of *deqi* with EEG microstate mapping on healthy subjects. Future clinical studies could be conducted on patients to further validate the marker value of EEG microstates and to provide more neuroimaging evidences for further explaining the therapeutic effects of acupuncture with *deqi*. In addition, although the *deqi* index could characterize the significant difference between different conditions, the behavior questionnaire could be further optimized. Fourth, only the basic acupuncture unit, the Hegu acupoint, was used as an example to explore the neuromodulation effects of acupuncture with *deqi*. Future studies should further explore the neuromodulation mechanism by EEG microstates with other acupoints. Finally, magnetoencephalography (MEG) experiments could further add more evidence to explain the dynamic processing characteristics of the brain network during acupuncture manipulation.

## Conclusion

Despite acupuncture’s public acceptance and long history, how acupuncture manipulation modulates the human brain remains largely unknown. By using EEG microstates theory and multichannel EEG recordings under acupuncture manipulation and tactile/rest controls, our results showed that EEG microstates could be used as objective markers to reveal the neuromodulation effects of acupuncture with *deqi*. Compared with that of controls, acupuncture manipulation could significantly increase the duration, occurrence, and contribution parameters of microstate C, whereas it could decrease those parameters of microstate D. Besides, acupuncture manipulation could also significantly improve the transition probabilities with microstate C as node, whereas it could reduce the transition probabilities with microstate D as node. Moreover, the microstate parameters (microstate C’s duration and microstate B→C’s transition probability) showed a significantly positive correlation with acupuncture *deqi* behavior scores. Taken together, EEG microstates could be effective markers to provide novel evidence for explaining acupuncture’s neuromodulation effects on human central nervous system, which also contributes to the objectification of TCM.

## Data Availability Statement

The data and codes that support the findings of this study are available upon request.

## Ethics Statement

The studies involving human participants were reviewed and approved by the Institutional Review Board and Ethics Committee of Tianjin University. The patients/participants provided their written informed consent to participate in this study.

## Author Contributions

XS designed and conceptualized the research. KZ, LZ, and XS collected the data. SH, YS, JY, LZ, and XS analyzed the data. XS and SH wrote the manuscript. XS and DM contributed to supervision. All authors contributed to the article and approved the submitted version.

## Conflict of Interest

The authors declare that the research was conducted in the absence of any commercial or financial relationships that could be construed as a potential conflict of interest.

## Publisher’s Note

All claims expressed in this article are solely those of the authors and do not necessarily represent those of their affiliated organizations, or those of the publisher, the editors and the reviewers. Any product that may be evaluated in this article, or claim that may be made by its manufacturer, is not guaranteed or endorsed by the publisher.

## Footnotes

1
http://www.thomaskoenig.ch/index.php/software/microstates-in-eeglab

## References

[B1] Abreu R. Jorge J. Leal A. Koenig T. Figueiredo P. (2021). EEG microstates predict concurrent fMRI dynamic functional connectivity states. *Brain Topogr.* 34 41–55. 10.1007/s10548-020-00805-1 33161518

[B2] Andersson S. Lundeberg T. (1995). Acupuncture–from empiricism to science: functional background to acupuncture effects in pain and disease. *Med. Hypotheses* 45 271–281. 10.1016/0306-9877(95)90117-5 8569551

[B3] Aroxa F. H. D. A. Gondim I. T. G. D. O. Santos E. L. W. Coriolano M. D. G. W. D. S. Asano A. G. C. Asano N. M. J. (2017). Acupuncture as adjuvant therapy for sleep disorders in Parkinson’s disease. *J. Acupunct. Meridian. Stud.* 10 33–38. 10.1016/j.jams.2016.12.007 28254099

[B4] Asghar A. U. R. Green G. Lythgoe M. F. Lewith G. MacPherson H. (2010). Acupuncture needling sensation: the neural correlates of deqi using fMRI. *Brain Res.* 1315 111–118. 10.1016/j.brainres.2009.12.019 20025853

[B5] Baernighausen T. Tugwell P. Rottingen J.-A. Shemilt I. Rockers P. Geldsetzer P. (2017). Quasi-experimental study designs series-paper 4: uses and value. *J. Clin. Epidemiol.* 89 21–29. 10.1016/j.jclinepi.2017.03.012 28365303

[B6] Bassett D. S. Sporns O. (2017). Network neuroscience. *Nat. Neurosci.* 20 353–364. 10.1038/nn.4502 28230844 PMC5485642

[B7] Bian H.-R. Wang J. Han C.-X. Deng B. Wei X.-L. Che Y.-Q. (2011). Features extraction from EEG signals induced by acupuncture based on the complexity analysis. *Acta Physica Sinica* 60 118701–118701. 10.7498/aps.60.118701

[B8] Brandeis D. Naylor H. Halliday R. Callaway E. Yano L. (1992). Scopolamine effects on visual information processing, attention, and event-related potential map latencies. *Psychophysiology* 29 315–336. 10.1111/j.1469-8986.1992.tb01706.x 1626042

[B9] Britz J. Van De Ville D. Michel C. M. (2010). BOLD correlates of EEG topography reveal rapid resting-state network dynamics. *Neuroimage* 52 1162–1170. 10.1016/j.neuroimage.2010.02.052 20188188

[B10] Brodbeck V. Kuhn A. von Wegner F. Morzelewski A. Tagliazucchi E. Borisov S. (2012). EEG microstates of wakefulness and NREM sleep. *Neuroimage* 62 2129–2139. 10.1016/j.neuroimage.2012.05.060 22658975

[B11] Chae Y. Chang D.-S. Lee S.-H. Jung W.-M. Lee I.-S. Jackson S. (2013). Inserting needles into the body: a meta-analysis of brain activity associated with acupuncture needle stimulation. *J. Pain* 14 215–222. 10.1016/j.jpain.2012.11.011 23395475

[B12] Chen A. C. N. Liu F. J. Wang L. Arendt-Nielsen L. (2006). Mode and site of acupuncture modulation in the human brain: 3D (124-ch) EEG power spectrum mapping and source imaging. *Neuroimage* 29 1080–1091. 10.1016/j.neuroimage.2005.08.066 16325429

[B13] Chu C. Wang X. Cai L. Zhang L. Wang J. Liu C. (2020). Spatiotemporal EEG microstate analysis in drug-free patients with Parkinson’s disease. *Neuroimage Clin.* 25:102132. 10.1016/j.nicl.2019.102132 31884224 PMC6938947

[B14] Corbetta M. Shulman G. L. (2002). Control of goal-directed and stimulus-driven attention in the brain. *Nat. Rev. Neurosci.* 3 201–215. 10.1038/nrn755 11994752

[B15] De Clercq W. Vergult A. Vanrumste B. Van Hees J. Palmini A. Van Paesschen W. (2005). “A new muscle artifact removal technique to improve the interpretation of the ictal scalp electroencephalogram,” in *Proceeding of the 2005 27th Annual International Conference of the IEEE Engineering in Medicine and Biology Society*, 944–947. 10.1109/iembs.2005.1616571 17282340

[B16] Ernst E. (2006). Acupuncturea critical analysis. *J. Intern. Med.* 259 125–137. 10.1111/j.1365-2796.2005.01584.x 16420542

[B17] Faber P. L. Travis F. Milz P. Parim N. (2017). EEG microstates during different phases of transcendental meditation practice. *Cogn. Process.* 18 307–314. 10.1007/s10339-017-0812-y 28451913

[B18] Fang J. Jin Z. Wang Y. Li K. Kong J. Nixon E. E. (2009). The salient characteristics of the central effects of acupuncture needling: limbic-paralimbic-neocortical network modulation. *Hum. Brain Mapp.* 30 1196–1206. 10.1002/hbm.20583 18571795 PMC6871074

[B19] Fox M. D. Corbetta M. Snyder A. Z. Vincent J. L. Raichle M. E. (2006). Spontaneous neuronal activity distinguishes human dorsal and ventral attention systems. *Proc. Natl. Acad. Sci. U.S.A.* 103 10046–10051. 10.1073/pnas.0604187103 16788060 PMC1480402

[B20] Fu Y. Chen J. Xiong X. (2018). Calculation and analysis of microstate related to variation in executed and imagined movement of force of hand clenching. *Comput. Intell. Neurosci.* 15:9270685. 10.1155/2018/9270685 30224914 PMC6129787

[B21] Gao F. Jia H. Feng Y. (2018). Microstate and omega complexity analyses of the resting-state electroencephalography. *Jove J. Visual. Exp.* 136:56452. 10.3791/56452 29985306 PMC6101748

[B22] Gomez-Herrero G. De Clercq W. Anwar H. Kara O. Egiazarian K. Van Huffel S. (2006). “Automatic removal of ocular artifacts in the EEG without an EOG reference channel,” in *Proceedings of the 7th Nordic Signal Processing Symposium, NORSIG 2006*, 130–133. 10.1109/norsig.2006.275210

[B23] Hsu S.-F. Chen C.-Y. Ke M.-D. Huang C.-H. Sun Y.-T. Lin J.-G. (2011). Variations of brain activities of acupuncture to TE5 of left hand in normal subjects. *Am. J. Chin. Med.* 39 673–686. 10.1142/s0192415x11009111 21721148

[B24] Hsu S.-H. Pion-Tonachini L. Palmer J. Miyakoshi M. Makeig S. Jung T.-P. (2018). Modeling brain dynamic state changes with adaptive mixture independent component analysis. *Neuroimage* 183 47–61. 10.1016/j.neuroimage.2018.08.001 30086409 PMC6205696

[B25] Hui K. K. S. Liu J. Marina O. Napadow V. Haselgrove C. Kwong K. K. (2005). The integrated response of the human cerebro-cerebellar and limbic systems to acupuncture stimulation at ST 36 as evidenced by fMRI. *NeuroImage* 27 479–496. 10.1016/j.neuroimage.2005.04.037 16046146

[B26] Hui K. K. S. Nixon E. E. Vangel M. G. Liu J. Marina O. Napadow V. (2007). Characterization of the “deqi” response in acupuncture. *BMC Complement. Altern. Med.* 7:33. 10.1186/1472-6882-7-33 17973984 PMC2200650

[B27] Khanna A. Pascual-Leone A. Farzan F. (2014). Reliability of resting-state microstate features in electroencephalography. *PLoS One* 9:e114163. 10.1371/journal.pone.0114163 25479614 PMC4257589

[B28] Khanna A. Pascual-Leone A. Michel C. M. Farzan F. (2015). Microstates in resting-state EEG: current status and future directions. *Neurosci. Biobehav. Rev.* 49 105–113. 10.1016/j.neubiorev.2014.12.010 25526823 PMC4305485

[B29] Kikuchi M. Koenig T. Munesue T. Hanaoka A. Strik W. Dierks T. (2011). EEG microstate analysis in drug-naive patients with panic disorder. *PLoS One* 6:e22912. 10.1371/journal.pone.0022912 21829554 PMC3146502

[B30] Kim H. (2014). Involvement of the dorsal and ventral attention networks in oddball stimulus processing: a meta-analysis. *Hum. Brain Mapp.* 35 2265–2284. 10.1002/hbm.22326 23900833 PMC6868981

[B31] Koenig T. Brandeis D. (2016). Inappropriate assumptions about EEG state changes and their impact on the quantification of EEG state dynamics. *Neuroimage* 125 1104–1106. 10.1016/j.neuroimage.2015.06.035 26091853

[B32] Koenig T. Lehmann D. Merlo M. C. G. Kochi K. Hell D. Koukkou M. (1999). A deviant EEG brain microstate in acute, neuroleptic-naive schizophrenics at rest. *Eur. Arch. Psychiatry Clin. Neurosci.* 249 205–211. 10.1007/s004060050088 10449596

[B33] Koenig T. Prichep L. Lehmann D. Sosa P. V. Braeker E. Kleinlogel H. (2002). Millisecond by millisecond, year by year: normative EEG microstates and developmental stages. *Neuroimage* 16 41–48. 10.1006/nimg.2002.1070 11969316

[B34] Kong J. Fufa D. T. Gerber A. J. Rosman A. S. Vangel M. G. Gracely R. H. (2005). Psychophysical outcomes from a randomized pilot study of manual, electro, and sham acupuncture treatment on experimentally induced thermal pain. *J. Pain* 6 55–64. 10.1016/j.jpain.2004.10.005 15629419

[B35] Kong S. -p. Tan Q. -w. Liu Y. Jing X. -h. Zhu B. Huo Y. -j. (2015). Specific correlation between the Hegu Point (LI4) and the orofacial part: evidence from an fMRI study. *Evid. Based Complement. Alternat. Med.* 2015:585493. 10.1155/2015/585493 26446439 PMC4584065

[B36] Kucyi A. Hove M. J. Esterman M. Hutchison R. M. Valera E. M. (2017). Dynamic brain network correlates of spontaneous fluctuations in attention. *Cereb. Cortex* 27 1831–1840. 10.1093/cercor/bhw029 26874182 PMC6317462

[B37] Lehmann D. Faber P. L. Galderisi S. Herrmann W. M. Kinoshita T. Koukkou M. (2005). EEG microstate duration and syntax in acute, medication-naive, first-episode schizophrenia: a multi-center study. *Psychiatry Res. Neuroimag.* 138 141–156. 10.1016/j.pscychresns.2004.05.007 15766637

[B38] Lehmann D. Ozaki H. Pal I. (1987). EEG alpha map series: brain micro-states by space-oriented adaptive segmentation. *Electroencephalogr. Clin. Neurophysiol.* 67 271–288. 10.1016/0013-4694(87)90025-3 2441961

[B39] Lehmann D. Pascual-Marqui R. Michel C. (2009). EEG microstates. *Scholarpedia* 4:7632. 10.4249/scholarpedia.7632

[B40] Lehmann D. Skrandies W. (1980). Reference-free identification of components of checkerboard-evoked multichannel potential fields. *Electroencephalogr. Clin. Neurophysiol.* 48 609–621. 10.1016/0013-4694(80)90419-8 6155251

[B41] Liang P. Wang Z. Qian T. Li K. (2014). Acupuncture stimulation of taichong (Liv3) and hegu (LI4) modulates the default mode network activity in Alzheimer’s disease. *Am. J. Alzheimers Dis. Other Dementias* 29 739–748. 10.1177/1533317514536600 24906968 PMC10852898

[B42] Lukacz E. S. Lawrence J. M. Burchette R. J. Luber K. M. Nager C. W. Buckwalter J. G. (2004). The use of visual analog scale in urogynecologic research: a psychometric evaluation. *Am. J. Obstet. Gynecol.* 191 165–170. 10.1016/j.ajog.2004.04.047 15295359

[B43] Marder E. (2012). Neuromodulation of neuronal circuits: back to the future. *Neuron* 76 1–11. 10.1016/j.neuron.2012.09.010 23040802 PMC3482119

[B44] Menon V. (2011). Large-scale brain networks and psychopathology: a unifying triple network model. *Trends Cogn. Sci.* 15 483–506. 10.1016/j.tics.2011.08.003 21908230

[B45] Michel C. M. Koenig T. (2018). EEG microstates as a tool for studying the temporal dynamics of whole-brain neuronal networks: a review. *Neuroimage* 180 577–593. 10.1016/j.neuroimage.2017.11.062 29196270

[B46] Milz P. Faber P. L. Lehmann D. Koenig T. Kochi K. Pascual-Marqui R. D. (2016). The functional significance of EEG microstates-Associations with modalities of thinking. *Neuroimage* 125 643–656. 10.1016/j.neuroimage.2015.08.023 26285079

[B47] Miranda J. Lamana S. M. S. Dias E. V. Athie M. Parada C. A. Tambeli C. H. (2015). Effect of pain chronification and chronic pain on an endogenous pain modulation circuit in rats. *Neuroscience* 286 37–44. 10.1016/j.neuroscience.2014.10.049 25451282

[B48] Mishra A. Englitz B. Cohen M. X. (2020). EEG microstates as a continuous phenomenon. *Neuroimage* 208:116454. 10.1016/j.neuroimage.2019.116454 31841679

[B49] Murphy M. Whitton A. E. Deccy S. Ironside M. L. Rutherford A. Beltzer M. (2020). Abnormalities in electroencephalographic microstates are state and trait markers of major depressive disorder. *Neuropsychopharmacology* 45 2030–2037. 10.1038/s41386-020-0749-1 32590838 PMC7547108

[B50] Murray M. M. Brunet D. Michel C. M. (2008). Topographic ERP analyses: a step-by-step tutorial review. *Brain Topogr.* 20 249–264. 10.1007/s10548-008-0054-5 18347966

[B51] Musso F. Brinkmeyer J. Mobascher A. Warbrick T. Winterer G. (2010). Spontaneous brain activity and EEG microstates. A novel EEG/fMRI analysis approach to explore resting-state networks. *Neuroimage* 52 1149–1161. 10.1016/j.neuroimage.2010.01.093 20139014

[B52] Nicolaou N. Georgiou J. (2012). Detection of epileptic electroencephalogram based on permutation entropy and support vector machines. *Exp. Syst. Appl.* 39 202–209. 10.1016/j.eswa.2011.07.008

[B53] Nishida K. Morishima Y. Yoshimura M. Isotani T. Irisawa S. Jann K. (2013). EEG microstates associated with salience and frontoparietal networks in frontotemporal dementia, schizophrenia and Alzheimer’s disease. *Clin. Neurophysiol.* 124 1106–1114. 10.1016/j.clinph.2013.01.005 23403263

[B54] Onton J. Delorme A. Makeig S. (2005). Frontal midline EEG dynamics during working memory. *Neuroimage* 27 341–356. 10.1016/j.neuroimage.2005.04.014 15927487

[B55] Palaniyappan L. Liddle P. F. (2012). Does the salience network play a cardinal role in psychosis? An emerging hypothesis of insular dysfunction. *J. Psychiatry Neurosci.* 37 17–27. 10.1503/jpn.100176 21693094 PMC3244495

[B56] Paraskeva A. Melemeni A. Petropoulos G. Siafaka I. Fassoulaki A. (2004). Needling of the extra 1 point decreases BIS values and preoperative anxiety. *Am. J. Chin. Med.* 32 789–794. 10.1142/s0192415x04002363 15633813

[B57] Pedroni A. Gianotti L. R. R. Koenig T. Lehmann D. Faber P. Knoch D. (2017). Temporal characteristics of EEG microstates mediate trial-by-trial risk taking. *Brain Topogr.* 30 149–159. 10.1007/s10548-016-0539-6 27933418

[B58] Schumacher J. Peraza L. R. Firbank M. Thomas A. J. Kaiser M. Gallagher P. (2019). Dysfunctional brain dynamics and their origin in Lewy body dementia. *Brain* 142 1767–1782. 10.1093/brain/awz069 30938426 PMC6536851

[B59] Seitzman B. A. Abell M. Bartley S. C. Erickson M. A. Bolbecker A. R. Hetrick W. P. (2017). Cognitive manipulation of brain electric microstates. *Neuroimage* 146 533–543. 10.1016/j.neuroimage.2016.10.002 27742598 PMC5321823

[B60] Skrandies W. (1989). Data reduction of multichannel fields: global field power and principal component analysis. *Brain Topogr.* 2 73–80. 10.1007/bf01128845 2641477

[B61] Smith S. M. Fox P. T. Miller K. L. Glahn D. C. Fox P. M. Mackay C. E. (2009). Correspondence of the brain’s functional architecture during activation and rest. *Proc. Natl. Acad. Sci. U.S.A.* 106 13040–13045. 10.1073/pnas.0905267106 19620724 PMC2722273

[B62] Strelets V. Faber P. L. Golikova J. Novototsky-Vlasov V. Koenig T. Gianotti L. R. R. (2003). Chronic schizophrenics with positive symptomatology have shortened EEG microstate durations. *Clin. Neurophysiol.* 114 2043–2051. 10.1016/s1388-2457(03)00211-6 14580602

[B63] Takahashi T. (2011). Mechanism of acupuncture on neuromodulation in the gut-a review. *Neuromodulation* 14 8–12. 10.1111/j.1525-1403.2010.00295.x 21992155

[B64] Tomescu M. I. Rihs T. A. Becker R. Britz J. Custo A. Grouiller F. (2014). Deviant dynamics of EEG resting state pattern in 22q11.2 deletion syndrome adolescents: a vulnerability marker of schizophrenia? *Schizophr. Res.* 157 175–181. 10.1016/j.schres.2014.05.036 24962438

[B65] Tyvaert L. LeVan P. Dubeau F. Gotman J. (2009). Noninvasive dynamic imaging of seizures in epileptic patients. *Hum. Brain Mapp.* 30 3993–4011. 10.1002/hbm.20824 19507156 PMC3767605

[B66] Uddin L. Q. (2015). Salience processing and insular cortical function and dysfunction. *Nat. Rev. Neurosci.* 16 55–61. 10.1038/nrn3857 25406711

[B67] Van de Ville D. Britz J. Michel C. M. (2010). EEG microstate sequences in healthy humans at rest reveal scale-free dynamics. *Proc. Natl. Acad. Sci. U.S.A.* 107 18179–18184. 10.1073/pnas.1007841107 20921381 PMC2964192

[B68] White P. Lewith G. Hopwood V. Prescott P. (2003). The placebo needle, is it a valid and convincing placebo for use in acupuncture trials? A randomised, single-blind, cross-over pilot trial. *Pain* 106 401–409. 10.1016/j.pain.2003.08.013 14659523

[B69] Wu M. T. Hsieh J. C. Xiong J. Yang C. F. Pan H. B. Chen Y. C. I. (1999). Central nervous pathway for acupuncture stimulation: localization of processing with functional MR imaging of the brainpreliminary experience. *Radiology* 212 133–141. 10.1148/radiology.212.1.r99jl04133 10405732

[B70] Xu J. Pan Y. Zhou S. Zou G. Liu J. Su Z. (2020). EEG microstates are correlated with brain functional networks during slow -wave sleep. *Neuroimage* 215:116786. 10.1016/j.neuroimage.2020.116786 32276057

[B71] Yu D. T. W. Jones A. Y. M. Pang M. Y. C. (2012). Development and validation of the chinese version of the massachusetts general hospital acupuncture sensation scale: an exploratory and methodological study. *Acupunct. Med.* 30 214–221. 10.1136/acupmed-2012-010145 22617434

[B72] Yu H. Liu J. Cai L. Wang J. Cao Y. Hao C. (2017). Functional brain networks in healthy subjects under acupuncture stimulation: an EEG study based on nonlinear synchronization likelihood analysis. *Physica Statist. Mech. Its Appl.* 468 566–577. 10.1016/j.physa.2016.10.068

[B73] Yu H. Wu X. Cai L. Deng B. Wang J. (2018). Modulation of spectral power and functional connectivity in human brain by acupuncture stimulation. *IEEE Trans. Neural Syst. Rehabil. Eng.* 26 977–986. 10.1109/tnsre.2018.2828143 29752232

[B74] Zhang Z.-J. Wang X.-M. McAlonan G. M. (2012). Neural acupuncture unit: a new concept for interpreting effects and mechanisms of acupuncture. *Evid. Based Complement. Alternat. Med.* 2012:429412. 10.1155/2012/429412 22474503 PMC3310280

