# Supplementary material for: The Temporal Dynamics of EEG Microstate Reveals the Neuromodulation Effect of Acupuncture With Deqi
Source: Front Neurosci. 2021 Oct 7;15:715512. doi: 10.3389/fnins.2021.715512 (PMC8549605; doi:10.3389/fnins.2021.715512)
Supplement: Supplementary file 1 [file Data_Sheet_1.pdf]

Supplementary Materials

The temporal dynamics of EEG microstate reveals the neuromodulation effect of acupuncture with *deqi*

Supplementary information	The details in Materials
Supplementary Figure S1	Correlation analysis between neural responses and acupuncture's behavior performances across all subjects
Supplementary Figure S2	Statistical comparisons of the transition probability
Supplementary Table S1	Subject information
Supplementary Table S2	Each subject's *deqi* behavior scores for acupuncture manipulation and tactile controls

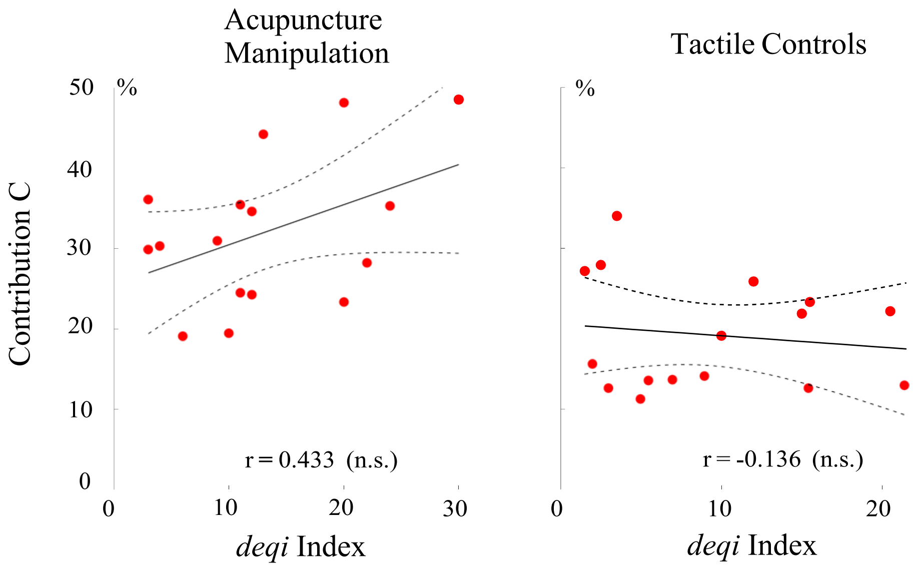

Supplementary Figure S1. Correlation analysis between neural responses and acupuncture's behavior performances across all subjects. Pearson's correlations between the contribution parameter of microstate C and the *deqi* behavior index for the acupuncture manipulation and tactile controls, respectively. Each red dot is indicated for each subject's data. Dash line the 95% confidence intervals for the mean of polynomial evaluation. Solid lines represent linear regression. (*p <0.05, N=16)

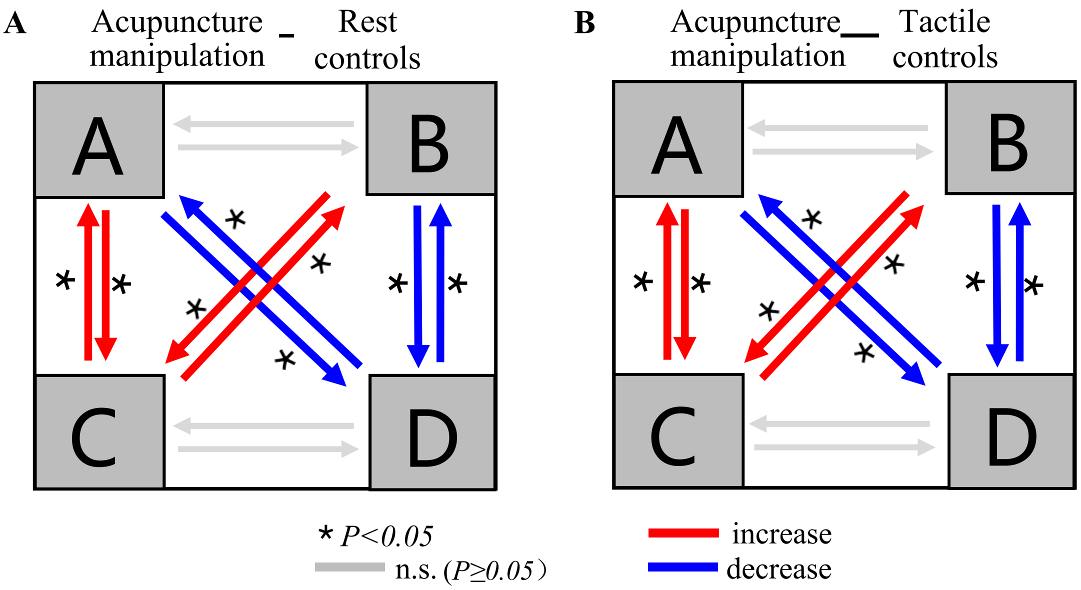

Supplementary Figure S2. Statistical comparisons of the transition probability for acupuncture manipulation (n=16) vs. rest controls (combining two rest controls, n=32) **(A)**, and for acupuncture manipulation (n=16) vs. tactile controls (combining two tactile controls, n=32) **(B)**. (Two sample t-tests with Bonferroni-correction for multiple comparisons, *p <0.05; the number of multiple comparisons of Bonferroni is 12).

Supplementary Table S1. Subject information.

Subject	Gender	Age(yrs)	Acupuncture site
1	Female	23	Hegu (LI4)
2	Female	23	Hegu (LI4)
3	Female	26	Hegu (LI4)
4	Male	22	Hegu (LI4)
5	Female	23	Hegu (LI4)
6	Male	23	Hegu (LI4)
7	Male	24	Hegu (LI4)
8	Male	22	Hegu (LI4)
9	Female	23	Hegu (LI4)
10	Female	24	Hegu (LI4)
11	Male	23	Hegu (LI4)
12	Male	27	Hegu (LI4)
13	Male	24	Hegu (LI4)
14	Male	25	Hegu (LI4)
15	Male	25	Hegu (LI4)
16	Male	28	Hegu (LI4)

Note: LI4=large intestine 4 (Hegu).

Supplementary Table S2. Each subject's *deqi* behavior scores for acupuncture manipulation and tactile controls.

Subject	Condition	Soreness	Numbness	Distention	Heaviness	Spread	Dull Pain	*deqi* index
1	Control(pre)	2	0	2	0	2	0	6
	Acupuncture	3	2	5	3	8	1	22
	Control(post)	2	4	3	3	5	1	18
2	Control(pre)	1	0	2	2	0	1	6
	Acupuncture	1	0	1	1	0	0	3
	Control(post)	0	0	1	0	0	0	1
3	Control(pre)	2	4	1	0	7	1	15
	Acupuncture	1	5	1	0	4	1	12
	Control(post)	2	5	1	0	6	1	15
4	Control(pre)	0	2	5	0	0	0	7
	Acupuncture	0	5	3	0	0	1	9
	Control(post)	0	2	2	0	0	0	4
5	Control(pre)	2	4	4	2	5	4	21
	Acupuncture	5	3	5	3	5	3	24
	Control(post)	3	4	5	3	4	3	22
6	Control(pre)	0	0	2	0	0	0	2
	Acupuncture	4	0	0	0	0	0	4
	Control(post)	2	0	0	0	0	0	2
7	Control(pre)	3	3	4	3	4	4	21
	Acupuncture	5	4	5	5	5	6	30
	Control(post)	3	3	4	3	4	3	20
8	Control(pre)	2	0	5	0	6	3	16
	Acupuncture	2	4	4	0	7	3	20
	Control(post)	0	0	0	0	2	0	2
9	Control(pre)	0	0	3	0	0	0	3
	Acupuncture	0	0	4	0	4	2	10
	Control(post)	0	0	2	0	0	0	2
10	Control(pre)	0	0	0	0	0	2	2
	Acupuncture	0	2	2	0	0	2	6
	Control(post)	0	1	1	0	0	2	4
11	Control(pre)	4	2	4	2	0	2	14
	Acupuncture	5	4	2	2	2	5	20
	Control(post)	4	2	4	1	2	4	17
12	Control(pre)	1	1	2	1	1	1	7
	Acupuncture	3	2	2	2	2	2	13
	Control(post)	1	1	2	1	1	1	7
13	Control(pre)	0	0	0	0	0	0	0
	Acupuncture	3	0	4	2	0	3	12
	Control(post)	3	2	3	1	0	1	10
14	Control(pre)	2	3	2	1	5	2	15
	Acupuncture	3	3	1	1	2	1	11
	Control(post)	2	4	3	1	5	1	16
15	Control(pre)	3	0	0	0	0	0	3
	Acupuncture	1	0	0	0	0	2	3
	Control(post)	0	0	0	0	0	0	0
16	Control(pre)	1	1	2	2	3	1	10
	Acupuncture	1	3	3	1	1	2	11
	Control(post)	1	2	1	2	3	1	10

Note: Control (pre) = Pre-manipulation tactile control, Acupuncture = acupuncture manipulation, Control (prost) = Post-manipulation tactile control.
